# Egg White and Yolk Protein Atlas: New Protein Insights of a Global Landmark Food

**DOI:** 10.3390/foods12183470

**Published:** 2023-09-18

**Authors:** Eleana Sarantidi, Alexandra Ainatzoglou, Christine Papadimitriou, Eleni Stamoula, Katerina Maghiorou, Argyro Miflidi, Antonia Trichopoulou, Konstantinos C. Mountzouris, Athanasios K. Anagnostopoulos

**Affiliations:** 1 Department of Biotechnology, Center of Systems Biology, Biomedical Research Foundation of the Academy of Athens, 11527 Athens, Greece; 2 Laboratory of Nutritional Physiology and Feeding, Department of Animal Science, School of Animal Biosciences, Agricultural University of Athens, 11855 Athens, Greece; 3 Academy of Athens, 10679 Athens, Greece

**Keywords:** egg, chicken, proteins, proteomics, egg white, egg yolk

## Abstract

(1) Background: The chicken egg is an animal product of great agronomic interest. The egg white and yolk constitute high-quality protein sources for humans with high digestibility and well-balanced amino acid profiles. Despite the egg white and yolk protein’s undisputed value, research to unravel their full proteome content and its properties is still ongoing. We aimed to exhaustively analyze the proteome of egg white and yolk by applying intrinsic proteomics and bioinformatics approaches in order to unravel the full protein potential of this landmark food. (2) Methods: A total of 45 freshly laid, unfertilized, chicken eggs were subjected to nanoLC-MS/MS Orbitrap analysis following a peptide pre-fractionation step. A comprehensive bioinformatics processing step was undertaken towards elucidating potential activities and roles of identified molecules. In parallel, the literature was mined concerning all reported egg white and yolk protein identifications. (3) Results: Our analysis revealed 371 and 428 new proteins, reported for the first time to be present in the egg white and yolk, respectively. From the bioactivity standpoint, egg white and yolk proteins showed high enrichment for antioxidant and anti-inflammatory processes, while exerting high relevance for the apoptosis and focal adhesion pathways. (4) Conclusions: Egg white and yolk proteins exert diverse and multifaceted properties. A total of 799 proteins were reported for the first time as being part of the egg and yolk. Our novel protein data enriched those already published in the literature and the first ever chicken egg white and yolk Protein Atlas, comprising 1392 protein entries, was generated. This dataset will provide a cornerstone reference for future studies involving egg proteins.

## 1. Introduction

The hen’s (*Gallus gallus*) egg is a closed system, that is, a completely aseptic and self-sufficient biological entity. It comprises all the components and necessary nutrients required for embryonic development to safeguard the development of the chicken embryo without any possible means of contact with the mother after oviposition. Therefore, the avian egg white plays a pivotal role, serving as a protective barrier for the egg yolk by maintaining its position, absorbing potential vibrations and showcasing antimicrobial properties [1]. Furthermore, it is a rich source of protein, water and nutrients to cover the significant needs of the developing embryo. In fact, fresh raw eggs consist of water, protein, fat, ash and carbohydrates in proportions of 76.1%, 12.6%, 9.5%, 1.1%, and 0.7% (*w*/*w*), respectively [2]. These components are differentially allocated in the various compartments of the egg, such as the egg white, yolk, shell, vitelline membrane and chalazae. Protein content in the egg white is 10.9% *w*/*w* and 15.9% *w*/*w* in the egg yolk [2].

The chicken egg is an animal product of great agronomic interest, with a world production of 70.9 million tonnes in 2018 [3]. Egg protein is a source of biological value protein for humans due to its high digestibility and well-balanced amino acid composition. The chicken egg is a globally accepted food, free of religious and cultural barriers, which is widely consumed all over the world.

Apart from their biological features, it is well documented that eggs constitute a primary source of high-quality, largely bioavailable protein for the human diet, providing a standard for assessing the protein value of foods [4]. Furthermore, egg white and yolk proteins’ emulsifying, foaming and gelation capacities render them eligible for use as a raw material, being one of the most widespread ingredients in the food industry [1,5,6]. In tandem with their high nutritional value, another aspect that makes them a focal point for researchers is their multifaceted biological properties, including, among others, their antioxidant, anticancer and antimicrobial functions [7]. The latter functions are still far from being fully elucidated and merit extensive specialized research.

Proteomics offer a high analytical capacity allowing for in-depth identification of protein-rich biological materials and have been avidly applied in egg protein research since the early 2000s. The significance of these technologies in egg research is pivotal, and current challenges and shortcomings are mainly related to instrument sensitivity and method development issues, which, when alleviated, will allow the achievement of maximal identification rates. Knowledge on the egg white and yolk protein content has been gradually increasing; however, no study exists to unify all available knowledge towards a comprehensive egg protein dataset [8].

The aim of the study was at first to exhaustively analyze the proteome of egg white and yolk in-depth by applying an intrinsic proteomics approach and secondly, to use the generated data to enrich those already present in the literature to deliver the first ever egg white and yolk Protein Atlas dataset.

## 2. Materials and Methods

### 2.1. Database Screening

A systematic data collection was conducted using informative guidelines for systematic reviews and in accordance with the PRISMA statement (Preferred Reporting Items for Systematic Reviews and Meta-analyses) covering peer-reviewed studies included in the literature databases published until May 2023 [9,10]. The full range of published data regarding the egg proteome was investigated by scanning available bibliographies in the PubMed, Google Scholar and Scopus databases using the following search terms: (“egg proteome” OR “egg white/egg yolk proteomics” OR “proteomic techniques in egg” OR “egg bioactive proteins” OR “molecular function of egg proteome” OR “egg proteome database”) AND (“egg proteins nutritional value”). Factors taken under consideration during the literature screening process were the year of publication, the number of samples and protein identifications and the type of the proteomic technologies employed.

Following the literature screening, all available proteins and related information were extracted, and entries were deposited in a list constituting the egg white and egg yolk published proteome dataset. The latter dataset was enriched by the protein identifications delivered by the present study forging the first ever egg white and egg yolk Protein Atlas.

### 2.2. Sample Preparation

#### 2.2.1. Sample Collection

The *Gallus gallus* egg proteome may be affected by various factors such as avian genetics, hen age, hen diet, rearing conditions (free-range, cage or barn hens) and overall hen management. For the purpose of this study, 45 freshly laid, unfertilized, commercially available chicken eggs from the local market were selected randomly, including gross weight eggs from free-range, cage or barn hens. Subsequently, the eggshell was cracked, and the egg whites were manually separated from the egg yolks and homogenized with a magnetic stirrer until the viscosity was reduced. From this point onwards, the egg whites and the egg yolks were analyzed separately, after short-term storage at −20 °C.

#### 2.2.2. Protein Extraction

An overnight acetone precipitation was performed with a sample ratio of 1/3 *v*/*v*. Subsequently, the samples were centrifuged for 30 min at 2500× *g*, dried in a Speed Vac apparatus (Eppendorf, Hamburg, Germany) for 30 min, and 0.4 gr of the sample were dissolved in lysis buffer [4% w/w SDS, 0.1 M Tris-HCL, 0.1 M dithioerythritol (DTE) pH = 7.6]. A Bradford assay was performed to determine the protein concentration. According to the aforementioned assay, 200–500 μg of the sample were transferred to an Amicon Ultra 0.5 Centrifugal Filter Device (Merck, Darmstadt, Germany) and samples were centrifuged for 15 min at 13,000 rpm after the addition of 8 M urea solution; this procedure was performed twice. An alkylation step was carried out by iodoacetamide addition (0.05 M iodoacetamide in 8 M urea, 0.1 M Tris-HCL pH = 8.5, RT) and samples remained in the dark for 30 min. Lastly, digestion was carried out overnight (approx. 20 h) by addition of trypsin at RT (Roche Diagnostics GmbH, Mannheim, Germany) in a ratio of 1/100 enzyme to protein [11].

### 2.3. Peptide Fractionation

Three milligrams of peptide mixture from each sample were fractionated using a C-18 column (75 μm × 50 cm; 100 Å, 2-μm-bead-packed Acclaim PepMap RSLC, Thermo Scientific) on an Agilent 1200 nanoflow HPLC instrument (Agilent Technologies, Santa Clara, CA, USA) operated at 1 mL/min. Βuffer A consisted of 10 mM ammonium formate and buffer B consisted of 10 mM ammonium formate with 90% acetonitrile; both buffers were adjusted to pH 10.

Fractions were collected using a Dionex AFC-3000 fraction collector in a 96-deep-well plate at 1 min intervals. The gradient was as follows: 1% B to 25% B in 50 min, 60% B in 4 min, and ramped up to 70% B in 2 min.

The total number of fractions per sample concentrated in a Speed Vac apparatus was set to 14. Prior to concentration, ammonium hydroxide and/or ammonium formate were evaporated in a Speed Vac apparatus (Eppendorf, Hamburg, Germany).

### 2.4. LC-MS/MS Analysis

Peptide fractions 1–14 were subjected to nanoLC-MS/MS analysis. Initially, reversed-phase high-performance liquid chromatography (HPLC) took place using an analytical C-18 column (75 μm × 50 cm; 100 Å, 2 μm bead-packed Acclaim PepMap RSLC, Thermo Scientific), for the isolation and analysis of peptides, through an Ultimate-3000 system (Dionex, Thermo Scientific, Bremen, Germany) coupled to an LTQ-Velos Orbitrap Elite mass spectrometer (Thermo Scientific, Waltham, MA, USA). Data on HPLC gradient and MS conditions were reported previously [12]. Data-dependent MS/MS scans for the 20 most intense ions per sample scan were performed with higher-energy collision dissociation (HCD) fragmentation in the Orbitrap operated at a resolving power of 15,000 and a collision energy of 36 NSE%.

### 2.5. Bioinformatics Analysis

MS/MS spectra were assigned to proteins using the Proteome Discoverer (version 1.4.0.388) (Thermo Scientific). The data search was performed using the SEQUEST engine to conduct dataset matching for protein identification against the downloaded protein dataset for a *Gallus gallus* *.fasta database from UniProt (version 7/2022).

As is well known, the UniProt Knowledgebase—UniProtKB—is the central hub for the collection of functional information on proteins with accurate, consistent and rich annotations. UniProtKB consists of two sections: UniProtKB reviewed, also known as Swiss-Prot, with manually annotated records, to which data have been added by an expert bio-curation team of scientists, and UniProtKB unreviewed, also known as TrEMBL, with computationally annotated records that await manual annotation [13]. Data from the present study were searched only against the manually reviewed entries of UniProtKB, so that the highest quality of the data is ensured. The Proteome Discoverer parameters were used as described before by Stamoula et al. [14].

The lists of proteins created by Proteome Discoverer were exported to Microsoft Excel software for further data processing.

#### Bioactivity Classification

In terms of protein bioactivity, the peptides of the highly abundant proteins (score > 1.6) were searched against the BIOPEP-UWM database (https://biochemia.uwm.edu.pl/biopep-uwm/.org) (accessed on 29 May 2023) so that the proteins possessing antioxidant, antimicrobial, anti-hypertensive, anti-inflammatory or immunomodulatory properties, were registered. With regard to molecular function, the proteINSIDE database (https://www.proteinside.org/) (accessed on 29 May 2023) was used, so as to separate proteins into four categories according to their function: (1) transport, signal transduction and apoptosis, (2) metabolism, (3) oxidation and reduction, and (4) heat shock and chaperones. Further functional analyses of the total amount of the proteins identified by in-house experiments and published data were accomplished by using the gene ontology database (http://geneontology.org/) (accessed on 29 May 2023). Lastly, a Venn diagram was produced using the bioinformatics & evolutionary genomics platform (https://bioinformatics.psb.ugent.be/webtools/Venn/) (accessed on 29 May 2023).

## 3. Results

### 3.1. Studies Involving Protein Analysis of Egg White-Yolk Proteins

**Figure 1:**
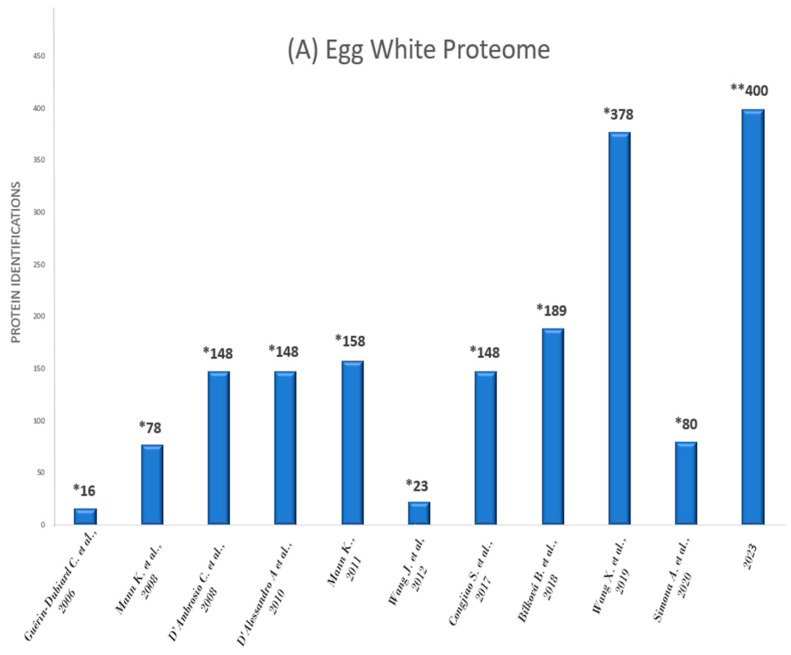

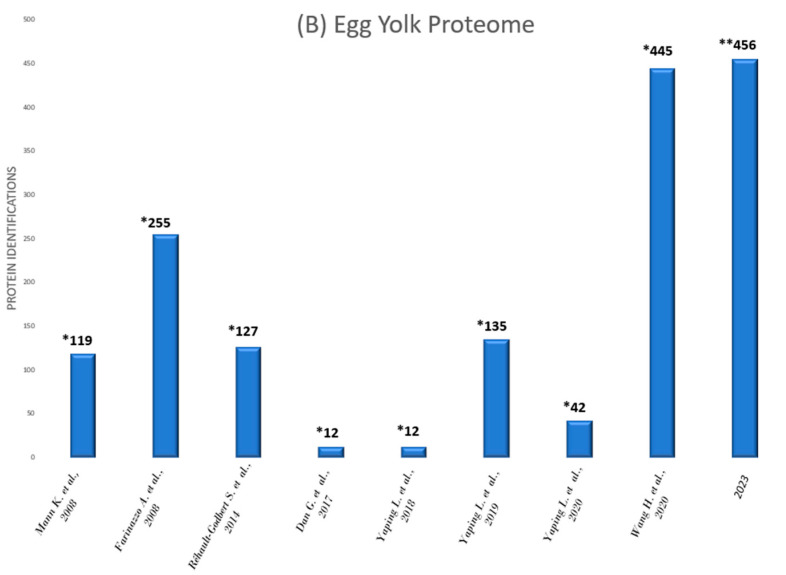
Timeline of proteomics data published across the years concerning egg white (**A**) and egg yolk (**B**) protein identifications. X axis: year of article publication. Y axis: number of protein identifications. * Indicates studies that used both reviewed & unreviewed protein datasets from the UniProt database. ** The present study used reviewed datasets of proteins from UniProt for MS/SM spectral identifications [15,16,17,18,19,20,21,22,23,24,25,26,27,28,29,30,31].

The first proteomic study on the egg white was conducted in 2006, which used a 2-DE MALDI-ToF MS approach and resulted in 16 protein identifications [15]. Corresponding studies on the egg yolk began two years later, using the same methodology, and reached a total of 119 protein identifications [16]. A sharp increase in protein annotations was noted in 2019 in a study from Wang X. et al. using an LC-MS/MS approach and identified 378 proteins in the egg white [17]. In 2020, the same group following the relevant experimental design increased the number of proteins identified in the egg yolk to 445 [18]. The present study, through an intrinsic experimental approach using an LC-MS/MS design, delivered the highest number of identifications reported, namely, 398 proteins for the egg white and 456 proteins for the egg yolk (Figure 1A,B).

A methodology leap concerning the number of identifications delivered by the present study compared to others, lies in the fact that, unlike previous publications, our MS/MS spectra analysis was based solely on the “Reviewed” UniProt protein database of *Gallus gallus* (Figure 1A,B).

### 3.2. Egg White and Egg Yolk Protein Bioactivity Classification

Egg proteins not only serve as nutrients, but are also implicated in the body’s physiological functions, and such functions are regulated by bioactive peptides [32]. A fundamental aim of the present study was to designate egg white and yolk proteins’ bioactive properties.

Following the methodology described in the previous section, protein identifications were assigned to five main categories of bioactivity: antioxidant, antimicrobial, antihypertensive, anti-inflammatory, and immunomodulatory.

**Figure 2:**
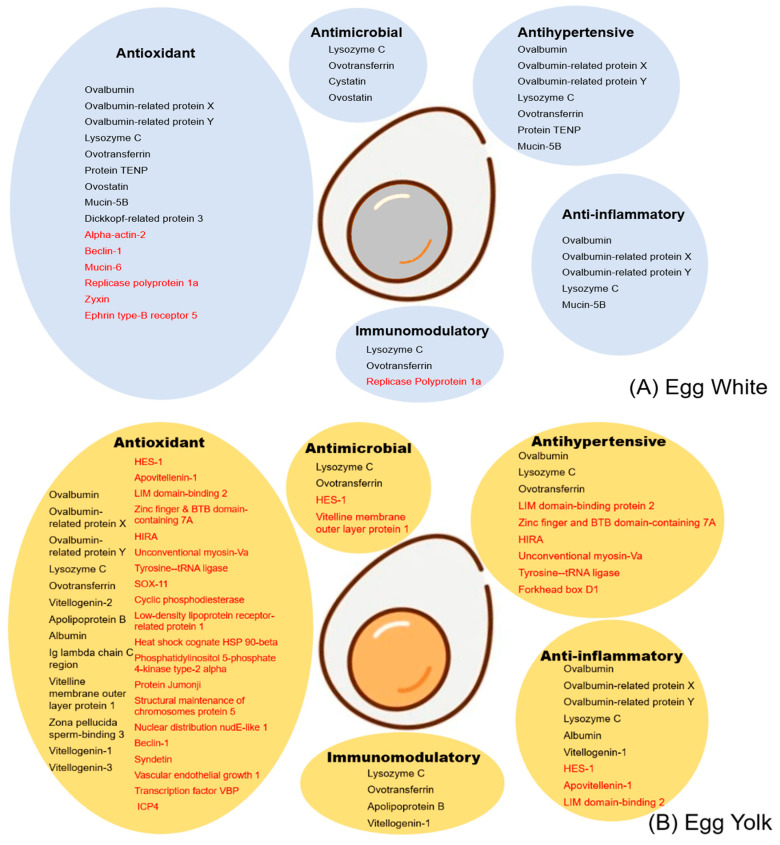
Grouping of proteins present in (**A**) egg white, (**B**) egg yolk according to their bioactivity. Molecules were assigned to five categories: antioxidant, antimicrobial, antihypertensive, anti-inflammatory, immunomodulatory. Protein names in black correspond to molecules reported by previous studies also identified by the present study. Protein names in red depict newly reported entries identified for the first time in the corresponding egg domains by the present study.

On this matter, egg white and egg yolk proteins showed higher enrichment regarding their antioxidant activity compared to the other categories. Key protein components, which are present in high abundance in the egg, like ovalbumin, lysozyme and ovotransferrin possess antioxidant characteristics. Also, antioxidant traits are exhibited by proteins identified for the first time by the present study, such as alpha actin-2 and becilin-1 in the egg white or HES-1 and apoviteline-1 in the egg yolk (Figure 2A,B).

Further, the proteins cystatin and ovostatin in part of the egg white as well as the vitelline membrane outer layer protein 1, which were identified for the first time by our study in the egg yolk, are characteristic examples of proteins with antimicrobial roles.

Another major bioactivity category was that of proteins known to have antihypertensive properties, examples of these are protein TENP and mucin-5B present in the egg white, whereas LIM domain-binding protein 2 and zinc finger & BTB domain-containing protein 7A are antihypertensive-related proteins identified for the first time in the egg yolk (Figure 2A,B).

Of note, several proteins such as ovalbumin and lysozyme in the egg white and HES-1 in the egg yolk are associated with anti-inflammatory action. Lastly, the main proteins implicated in immunomodulatory functions are lysozyme and ovotransferrin. A detailed view of the proteins present in the egg white and yolk grouped according to their relevant bioactivity functions is presented in Figure 2.

### 3.3. Molecular Function

A classification of proteins according to their molecular roles was performed as described in the Materials and Methods section and key categories used were: (1) transport, signal transduction and apoptosis, (2) metabolism, (3) oxidation and reduction, (4) heat shock and chaperones.

**Figure 3:**
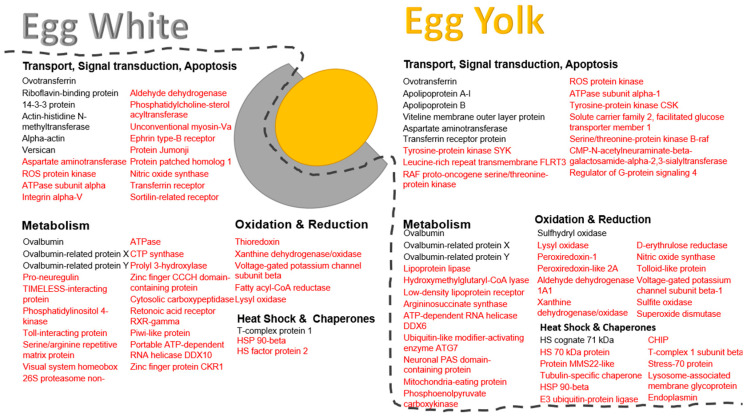
Overview of proteins present in the egg white and egg yolk categorized according to their molecular function. Protein names in black correspond to molecules reported by previous studies also identified by the present study. Protein names in red are newly reported entries identified for the first time in the corresponding egg domains by the present study.

Results showed that both egg white and yolk proteins were mostly enriched for the transport, signal transduction and apoptosis group (19 proteins in the egg white and 17 in the egg yolk); for example, the proteins ovotransferrin, riboflavin-binding protein and 14-3-3 protein gamma were key molecules in the category for egg white and in correspondence, ovotransferrin, apolipoprotein A-I and apolipoprotein B were key in the egg yolk category (Figure 3).

The second most prevalent molecular function was that of metabolism (20 proteins in the egg white and 13 in the egg yolk) including ovalbumin, ovalbumin-related protein X and ovalbumin-related protein Y, both for the egg white and for the egg yolk (Figure 3).

Of note, knowledge on the transport, signal transduction and apoptosis category was further supplemented by our study due to the addition of 13 new proteins including, for instance, ROS kinase, integrin alpha V and ephrin receptor present in egg white and 11 new proteins including tyrosine kinase, leucine-rich repeat transmembrane protein, and serine/threonine-protein kinase B-raf present in egg yolk. Accordingly, 17 new proteins were added to the metabolism category in the egg white (e.g., pro-neuregulin, phosphatidylinositol 4-kinase, serine/arginine repetitive matrix protein) and 10 new proteins (e.g., lipoprotein lipase, hydroxymethylglutaryl-CoA lyase, argininosuccinate synthase) in the egg yolk, respectively.

Interestingly, most proteins with an oxidative or reductive role and proteins with heat shock function, as well as chaperones, were newly identified and described for the first time by the current study. Specifically, five new entries (thioredoxin, xanthine dehydrogenase/oxidase, voltage-gated potassium channel subunit beta) were added to the egg white and thirteen (e.g., lysyl oxidase, peroxiredoxin-1, xanthine dehydrogenase/oxidase) to the egg yolk oxidation and reduction group, and two (HSP 90-beta, HS factor protein 2) new entries were incorporated in the egg white and ten (e.g., HS 70 kDa protein, protein MMS22-like, tubulin-specific chaperone) in the egg yolk in the heat shock proteins and chaperones category, respectively (Figure 3).

### 3.4. G0 Annotation and Enrichment Analysis for the Sum of Egg White and Egg Yolk Protein Identifications

GO annotation was performed using the Geneontology Resource platform (http://geneontology.org/) (accessed on 29 May 2023). Annotation analysis processing was performed using the sum of all protein identifications from all available studies (including the present study) as input for the egg white or yolk.

**Figure 4:**
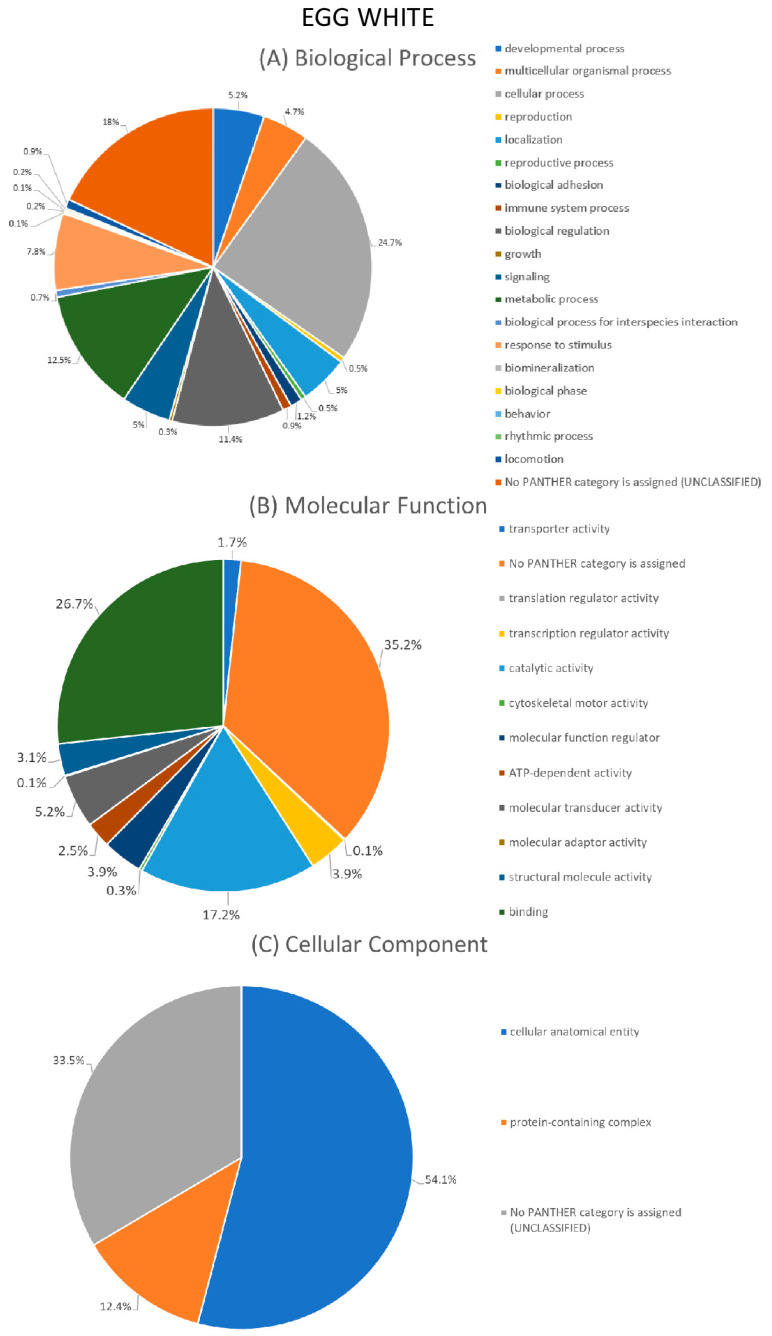
Gene Ontology (GO) annotation of the sum of egg white proteins available from all studies (including the present study) according to (**A**) biological process, (**B**) molecular function and (**C**) cellular component.

**Figure 5:**
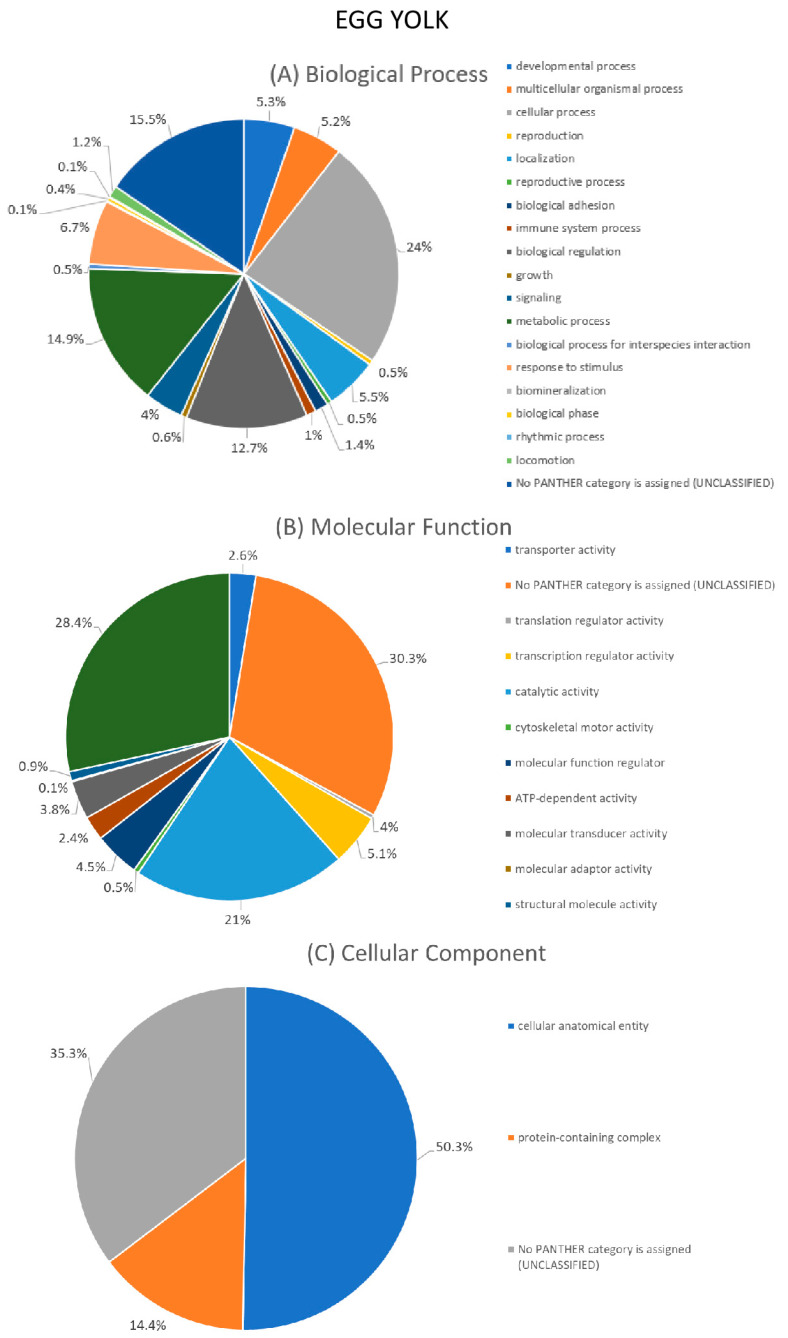
Gene Ontology (GO) annotation of the sum of egg yolk proteins available from all studies (including the present study) according to (**A**) biological process, (**B**) molecular function and (**C**) cellular component.

Classification of proteins took place for three major categories: biological process (BP), molecular function (MF) and cellular component (CC). As far as the BP grouping is concerned, the majority of proteins in the egg white (24.7%) and egg yolk (24%) were found to be involved in cellular processes (Figure 4A and Figure 5A). Other major categories were the metabolic process involving 12.5% of egg white and 14.9% of the egg yolk proteins and biological regulation with 11.4% of egg white and 12.7% of the egg yolk proteins, respectively.

Regarding the MF category, the majority of proteins in the egg white (26.7%) and the egg yolk (28.4%) were found to possess a binding function. Further, a total of 17.2% of proteins in the egg white and 21% in the egg yolk were assigned a catalytic activity. A total of 5.2% of egg white and 3.8% of egg yolk proteins were apportioned a molecular transducer activity (Figure 4B and Figure 5B).

Lastly, in the CC category, the majority of proteins, 54,1% and 50,3%, respectively, belong to the cellular anatomical entity, whereas 12.4% and 14.4%, respectively, belong to the protein-containing complex categories (Figure 4C and Figure 5C).

It is important to note that a large number of proteins, both in the BP, MF and CC integration, were not classifiable into any category and appear under «unclassified». This is because many of the protein inputs (derived from previous studies) are considered “unreviewed” in terms of their UniProt status and subsequently their function cannot be determined.

### 3.5. KEGG Annotation and Enrichment Analysis for the Sum of Egg White and Egg Yolk Proteins

Functional annotation of the sum of identified proteins (from all previous studies including the present study) for the egg white and egg yolk was performed by the Kyoto Encyclopedia of Genes and Genomes to elucidate the functions of identified molecules and elucidate the metabolic or signaling pathways they are involved in.

**Figure 6:**
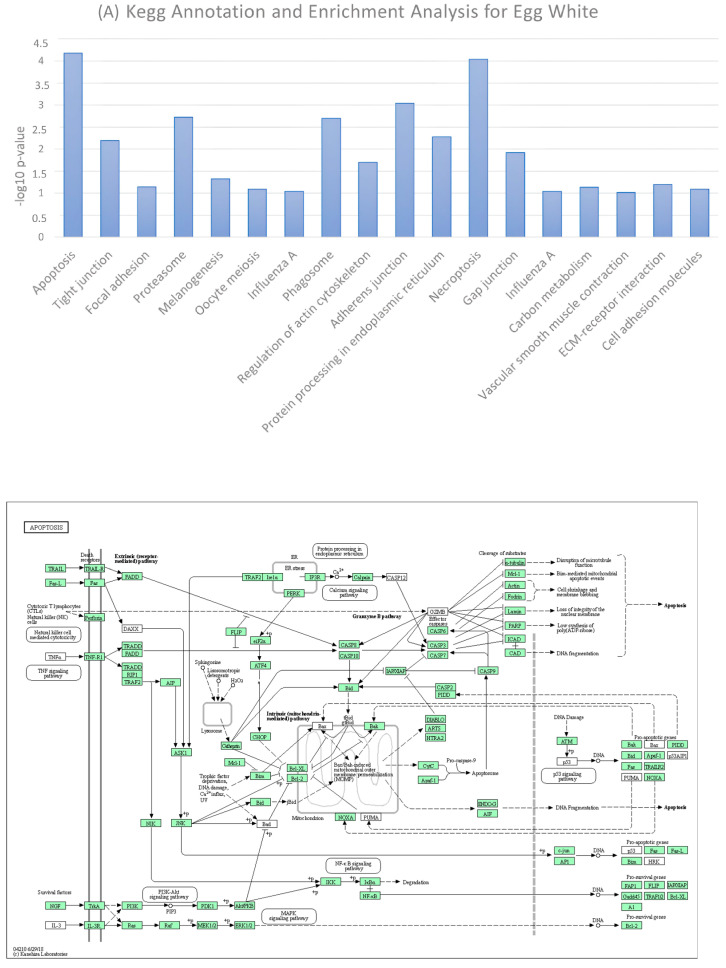
(**A**) KEGG pathway enrichment analysis for all identified proteins in egg white. The X— axis shows KEGG pathways significantly enriched for the identified proteins and the Y— axis shows the enrichment scores of these items. (**B**) Pathway map for identified proteins and their positioning in the apoptosis pathway. Solid line connects two pathway nodes if a path between them exists in the KEGG database. Dotted line indicates an inferred path from an input sequence or a query node to a known pathway.

The KEGG pathway showing the highest enrichment for all identified proteins in the egg white was the apoptosis pathway (Figure 6A). The specific function is defined as the genetically programmed process for the elimination of damaged or redundant cells by activation of caspases (aspartate-specific cysteine proteases) [33,34].

Protein hits for egg white associated with apoptosis were subjected to pathway enrichment analysis and the relevant schema showing the intercorrelation of identified molecules in the pathway is shown in Figure 6B.

**Figure 7:**
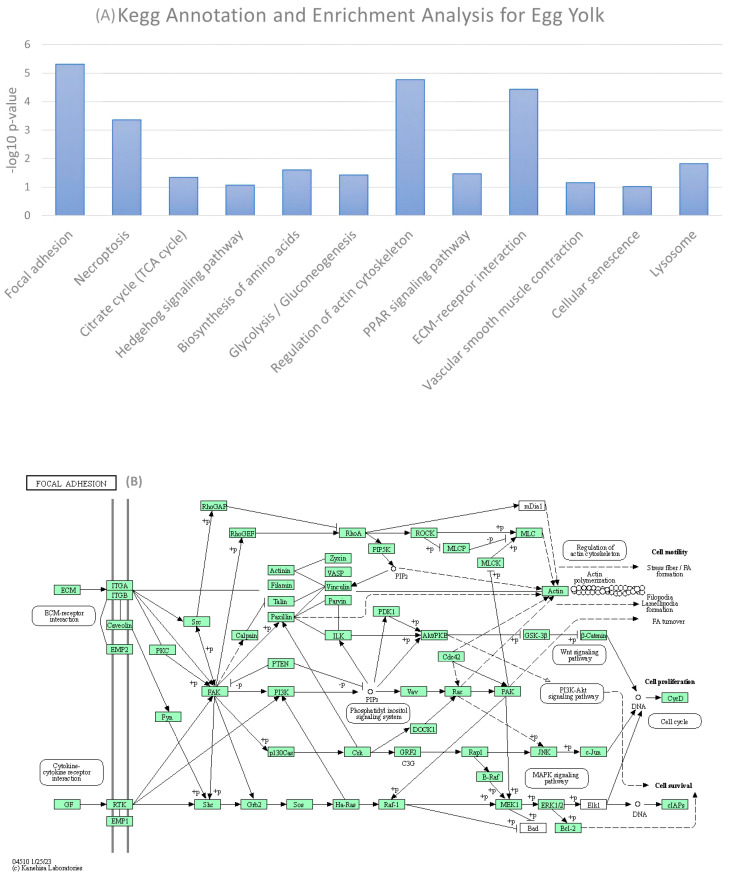
(**A**) KEGG pathway enrichment analysis for all identified proteins in egg yolk. The X—axis shows KEGG pathways significantly enriched for the identified proteins and the Y—axis shows the enrichment scores of these items. (**B**) Pathway map for identified proteins and their positioning in the focal adhesion pathway. Solid lines connect two pathway nodes if a path between them exists in the KEGG database. Dotted line indicates an inferred path from an input sequence or a query node to a known pathway.

Accordingly, a KEGG annotation diagram was conducted for the egg yolk, revealing that proteins showed the highest KEGG enrichment for the focal adhesion pathway. This connects egg yolk proteins with important biological processes including cell motility, cell proliferation, cell differentiation, regulation of gene expression and cell survival (Figure 7A). The proteins involved in the pathway and how they are interconnected are clearly represented on the pathway map presented in Figure 7B.

### 3.6. Egg White and Egg Yolk Protein Atlas

Proteome data from published articles concerning the egg white and egg yolk, including the results of the present study, were for the first time compiled and incorporated into an up-to-date dataset to form the total egg white and egg yolk proteome atlas (Supplementary Tables S1 and S2).

**Figure 8:**
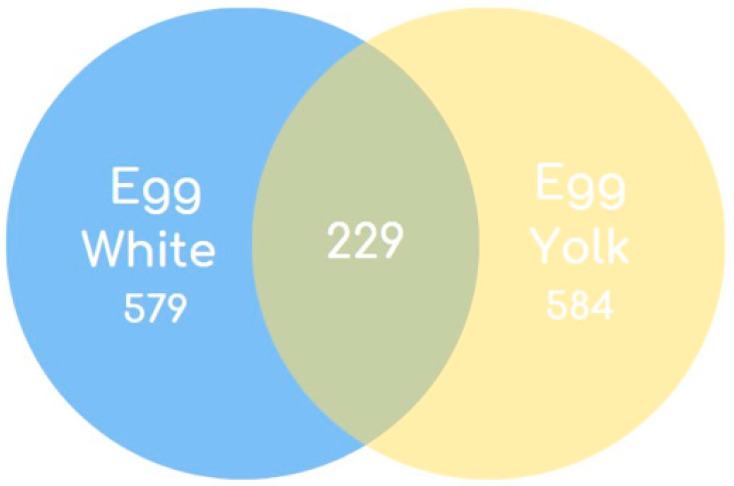
Egg white and egg yolk Protein Atlas. A total of 1392 unique proteins are present in the egg white and egg yolk. The figure presents data including identifications from all previously published articles as well as proteins reported in the present study. A Venn diagram is used to show that 579 proteins are uniquely present in the egg white, 584 proteins are characteristic for the egg yolk and 229 are commonly present in the egg white and egg yolk.

Specifically, a total of 808 egg white and 813 egg yolk proteins are registered in the dataset, out of which 579 proteins are present in the egg white only, 584 proteins are solely expressed in the egg yolk and 229 proteins are common in the two egg parts (Figure 8).

Of note, 371 proteins in the egg white and 428 proteins in the egg yolk are new identifications, registered for the first time by the present study (Table 1).

## 4. Discussion

The hen’s (*Gallus gallus*) egg is a closed system, comprising all the components and nutrients required for embryonic development. The egg white and yolk are a rich source of a variety of proteins that have emulsifying, foaming and gelation properties making them a useful raw material, whilst being a landmark food and one of the most prevalent ingredients in the human diet. The present study employed the analytical dynamics of proteomics to study the proteome of egg white and yolk in depth. Further, by incorporating the generated data into already published protein databases, we achieved the construction of the first ever egg white and egg yolk Protein Atlas.

The present study stated the presence of 400 proteins in the egg white and 456 proteins in the egg yolk, and 371 and 428 proteins, respectively, were newly identified molecules reported for the first time (Table 1).

Our proteomics data were subjected to bioinformatics analyses, revealing that with regard to bioactivity, the egg white and egg yolk proteins show high enrichment on their antioxidant activity. The second most prevalent category for the egg white and yolk protein bioactivity was that of anti-inflammatory action. Further, identified proteins in the present study were assessed regarding their molecular function. Analyses revealed that both egg white and yolk proteins were mostly enriched for the “transport, signal transduction and apoptosis” group and the second most prevalent molecular function group was that of “metabolism”.

Once the identified proteins of the study were incorporated in the dataset of already published proteins, the sum of protein identifications for the egg white and yolk proteins was subjected to GO annotation and enrichment analysis revealing that the majority of egg white and yolk proteins are involved in “cellular processes”, they possess “binding function” and are located in the “cellular anatomical entity”. Also, the KEGG annotation and enrichment analysis unveiled that the two main pathways incorporating the sum of identified proteins were the “apoptosis pathway” for the egg white and the “focal adhesion pathway” for the egg yolk.

Importantly, the present study provided novel knowledge on the bioactivity, molecular function and pathway distribution of the vast majority of egg white and yolk proteins identified to date. Proteins exert their bioactive role in the form of peptides. Proteins are cleaved into peptides through hydrolysis by proteases present in the human or animal gastrointestinal tract, such as trypsin, chymotrypsin and pepsin, or other plant and microbe-derived proteases, such as thermolysin, alcalase and papain, the exploration of which has expanded the potential of egg proteins [35].

The results presented herein can drive research of bioactive peptides naturally present in this staple food towards ameliorating human health. It is known that the major underlying pathways involved in the pathogenesis of numerous medical conditions are oxidative stress and chronically active inflammation. In an attempt to gain control of these processes, scientists have shifted their focus to the discovery of bioactive peptides derived from food products of high nutritional value, such as the egg. As established herein, the egg contains a wide spectrum of biologically active compounds with multifaceted capacities, such as antioxidant, anti-inflammatory, anti-cancerous, antimicrobial and anti-hypertensive properties [36,37].

Due to their unique functional and biological features, some egg-derived peptides can act as precursors for the production of higher quality bioactive peptides that are useful to the nutraceutical and pharmaceutical industries. After the peptides are processed by certain proteases, the obtained hydrolysate is evaluated for a given property through exposure to certain elements. For instance, to assess the antimicrobial capacity of the emerging peptides, they are exposed to bacteria and the minimum inhibition concentration (MIC) is measured. Similarly, when examining their antihypertensive capacity, peptides are evaluated for their capacity to downregulate renin or angiotensin-converting enzyme (ACE) activity or to stimulate NO production [38]; whereas, when testing for antioxidants, ion chelation, free-radical scavenging and the mitigation of lipid peroxidation are measured [39]. The ACE structure and function were thoroughly tested in a relevant study [40].

The antioxidant properties of numerous egg-derived peptides have been well characterized. Namely, phosvitin, a protein present in egg yolk, has demonstrated metal-chelating potential, and its hydrolyzed byproducts exhibited free-radical scavenging capacity [41,42]. Regarding the highly prevalent ovalbumin and ovotransferrin, their potent antioxidant activities have also been reported and are known to be reinforced upon hydrolysis [43,44]. In fact, peptides derived from the enzymatic hydrolysis of ovotransferrin by pepsin and thermolysin have also displayed antihypertensive effects, through inhibition of ACE, whereas the equivalent was observed for lysozyme hydrolysates [45]. The latter protein has a well-established bacteriolytic effect on a wide range of pathogens, which has been employed in the food industry to create a natural food preservative [46]. Ovalbumin byproducts have also been proven to be highly effective against resistant microbes, and the iron-binding capacity of ovotransferrin prevents bacterial growth by hindering their access to iron [47]. Similarly, other egg proteins with antimicrobial potential include phosvitin, avidin, ovomucin, ovomacroglobulin, cystatin and IgY [48].

Taking it one step further, anti-cancerous properties of egg proteins have also been displayed. Cystatin has been employed as an anti-cancerous agent, exhibiting inhibitory action on intracellular tumor-related processes mediated by cysteine proteases [49]. This downregulation of cysteine proteases has been found to confine tumor invasiveness and minimize the metastatic potential of mutated breast epithelium. Furthermore, inhibition of cathepsin activity mediated by cystatin acts against gastric tumor growth [50].

Another protein correlated with anti-cancerous activity is avidin. This protein displays high affinity for biotin, while reinforcing the antitumor potential of TNF-a by fivefold [51,52]. Similarly, lysozyme has demonstrated tumor-preventive effects in vivo, an outcome mediated by immune cell stimulation and enhanced tumor immunogenicity [53]. At the same time, this protein hinders metastatic activity, while containing cancer cell growth by interfering with their cell cycle [54]. Similar anti-cancerous effects have been displayed by phosvitin, a protein that, apart from its antioxidant capacities discussed above, hinders DNA damage and exerts cytotoxic effects on tumor cells [55]. In the same manner, ovomucin present in egg white also exerts toxicity against cancer cells, while hindering tumor angiogenesis [56]. Regarding ovotransferrin, its byproducts downregulate tumor cell proliferation, while its cytotoxicity against cancer cells is increased through hydrolysis [57]. Lastly, ovalbumin was found to exert the most potent anti-cancerous effects compared to other egg proteins after exposure to heat and subsequent denaturation [58].

In a nutshell, the anti-cancerous properties exerted by egg proteins and their hydrolysates are mediated through a variety of pathways, such as DNA damage prevention, decreased tumor cell growth and invasiveness, antimutagenic effects, increased cytotoxicity and apoptosis. By extension, egg proteins that have also been associated with immunomodulatory properties include phosvitin, IgY, livetin, lysozyme, cystatin, ovomucin and ovotransferrin [48]. These have been attributed to a variety of effects including regulation of cytokine release, modulation of phagocytic activity, metalloproteinase stimulation, increase of antibody production, immune cell activation, interaction with adhesion molecules and other inflammatory mediators and interference with the MAPK, ERK and NF-kb pathways [59].

Core functions of some of the newly detected egg proteins reported in our study are more thoroughly discussed. Replicase polyprotein 1a has been found to participate in viral RNA replication and expression processes [60]. This protein also hinders the translation of host genes at the ribosomal level and interferes with nuclear transfer systems. Furthermore, its interaction with host PHB proteins that safeguard mitochondrial functionality and counteract stress render replicase polyprotein 1a capable of affecting host cell survival and align with its immunomodulatory and antioxidant properties found in our study [61].

Another protein family present in our study is that of mucins, which are found in secretory cells and are mainly comprised of glycans. These glycans often possess binding sites for pathogens and alterations in their glycosylation patterns increase susceptibility to mucosal inflammation and penetration of pathogens. Following mucin exocytosis, these proteins absorb water and perform ion exchange resulting in mucus gel formation [62].

Beclin-1 is a protein involved in oncogenesis and neurodegenerative diseases, through the process of autophagy, whose reinforcement provides better tumor response to treatment and enhanced overall prognosis. Low beclin-1 hippocampal levels have also been involved in the pathogenesis of schizophrenia, as the consequent downregulated autophagy causes neuronal cell death to rise [63]. HIRA is a protein with largely undetermined functions that probably affects chromatin and histone metabolic pathways.

Zyxin is a protein that anchors actin cytoskeleton to the cellular membrane by interacting with actin binding proteins, while also mediating intercellular adhesion. This protein has been found to display autoregulatory patterns [64]. A-actinin-2 (ACTN2) connects actin and titin molecules at Z-discs, an action regulated by phospholipids, while also binding cardiac ion channels. When subject to mutation, this protein has been linked to the pathogenesis of hypertrophic cardiomyopathy and other cardiovascular abnormalities [65,66].

HES-1 acts as a transcriptional factor, suppressing the expression of certain genes that necessitate the presence of basic helix–loop–helix proteins. This factor affects cell proliferation at various stages of embryonic development. Its involvement in neural stem cell differentiation has also been suggested, as its levels fluctuate across the CNS [67]. HES-1 also interacts with the Notch pathway, binding a Notch ligand that promotes neural differentiation. This binding results in a blockage of neural differentiation that sustains neural stem and progenitor cell populations [68]. Similarly, HES-1 exerts such effects in the GI tract, regulating pancreatic exocrine cell differentiation, as well as hepatocyte regeneration and bile duct formation [69]. SOX11 also contributes to nervous system development, neurogenesis and oncogenesis, as its mutations have been associated with the emergence of mantle cell lymphoma [70].

Jumonji is a protein that also acts as a transcriptional factor, as its presence in the nucleus is imperative for mouse embryogenesis, mediating processes such as DNA binding and transcriptional suppression via histone post-translational modifications [71]. Its contribution to organogenesis mainly lies in the development of neural, hepatic and cardiovascular system components, and its mutations lead to corresponding genetic abnormalities [72]. Moreover, disrupted expression of this protein has been linked with tumorigenesis in the colon, breast and prostate gland, as well as being correlated with hematological malignancies. Lastly, tyrosine tRNA ligase is a synthetase that binds amino acids to their corresponding tRNAs safeguarding their connections [73].

## 5. Conclusions

The hen egg, beyond its exceptional nutritional value, merits extensive research to unearth and understand its full-scale bioactive properties. The properties exerted by the egg white and egg yolk proteins and their peptides are diverse and multifaceted. This study contributed new basic knowledge of the egg proteome by identifying 371 new proteins in the egg white and 428 new proteins as part of the egg yolk. These proteins are predicted to chiefly have antioxidant and anti-inflammatory roles. Furthermore, KEGG pathway analyses revealed key implications of egg proteins in the apoptosis and focal adhesion pathways. Lastly, we generated the first ever egg white and egg yolk Protein Atlas comprising a total of 1392 protein entries, providing a cornerstone reference for future studies involving egg proteins.

## Figures and Tables

**Table 1:** Protein identifications concerning the egg white and egg yolk showing data from previous work and the present study.

	Egg White	Egg Yolk
Proteins identified in present study *	400	456
Unique proteins identified in present study (new entries)	371	428
Proteins identified in previous studies	408	357
Total proteins (all articles, incl. present study)	808	813

* A total of *n* = 45 egg white and *n* = 45 egg yolk samples were analyzed. Each sample was run in triplicates.

## Data Availability

The data used to support the findings of this study can be made available by the corresponding author upon request.

## Supplementary Materials

The following supporting information can be downloaded at: https://www.mdpi.com/article/10.3390/foods12183470/s1, Table S1: Egg White Protein Atlas; Table S2: Egg Yolk Protein Atlas.
